# Molecular signature of response to preoperative radiotherapy in locally advanced breast cancer

**DOI:** 10.1186/s13014-018-1129-4

**Published:** 2018-10-01

**Authors:** Miljana Tanić, Ana Krivokuća, Milena Čavić, Jasmina Mladenović, Vesna Plesinac Karapandžić, Stephan Beck, Siniša Radulović, Snezana Susnjar, Radmila Janković

**Affiliations:** 1 Laboratory for Molecular Genetics, Institute of Oncology and Radiology of Serbia, Belgrade, Serbia; 2 Radiology and Radiotherapy Department, Institute of Oncology and Radiology of Serbia, Belgrade, Serbia; 3 Medical Genomics, UCL Cancer Institute, University College London, London, UK; 4 Medical Oncology Department, Institute of Oncology and Radiology of Serbia, Belgrade, Serbia

**Keywords:** Preoperative radiotherapy, Locally advanced breast cancer, Biomarker, Gene expression profiling

## Abstract

**Background:**

Radiation therapy is an indispensable part of various treatment modalities for breast cancer. Specifically, for non-inflammatory locally advanced breast cancer (LABC) patients, preoperative radiotherapy (pRT) is currently indicated as a second line therapy in the event of lack of response to neoadjuvant chemotherapy. Still approximately one third of patients fails to respond favourably to pRT. The aim of this study was to explore molecular mechanisms underlying differential response to radiotherapy (RT) to identify predictive biomarkers and potential targets for increasing radiosensitivity.

**Methods:**

The study was based on a cohort of 134 LABC patients, treated at the Institute of Oncology and Radiology of Serbia (IORS) with pRT, without previous or concomitant systemic therapy. Baseline transcriptional profiles were established using Agilent 60 K microarray platform in a subset of 23 formalin-fixed paraffin-embedded (FFPE) LABC tumour samples of which 11 radiotherapy naïve and 3 post-radiotherapy samples passed quality control and were used for downstream analysis. Biological networks and signalling pathways underlying differential response to RT were identified using Ingenuity Pathways Analysis software. Predictive value of candidate genes in the preoperative setting was further validated by qRT-PCR in an independent subset of 60 LABC samples of which 42 had sufficient quality for data analysis, and in postoperative setting using microarray data from 344 node-negative breast cancer patients (Erasmus cohort, GSE2034 and GSE5327) treated either with surgery only (20%) or surgery with RT (80%).

**Results:**

We identified 192 significantly differentially expressed genes (FDR < 0.10) between pRT-responsive and non-responsive tumours, related to regulation of cellular development, growth and proliferation, cell cycle control of chromosomal replication, glucose metabolism and NAD biosynthesis II route. *APOA1*, *MAP3K4*, and *MMP14* genes were differentially expressed (FDR < 0.20) between pRT responders and non-responders in preoperative setting, while *MAP3K4* was further validated as RT-specific predictive biomarker of distant metastasis free survival (HR = 2.54, [95%CI:1.42–4.55], *p* = 0.002) in the postoperative setting.

**Conclusions:**

This study pinpoints *MAP3K4* as a putative biomarker of response to RT in both preoperative and postoperative settings and a potential target for radiosensitising combination therapy, warranting further pre-clinical studies and prospective clinical validation.

**Electronic supplementary material:**

The online version of this article (10.1186/s13014-018-1129-4) contains supplementary material, which is available to authorized users.

## Background

Non-inflammatory locally advanced breast carcinoma (LABC) is a late stage breast cancer presented as a bulky primary chest wall tumour and/or extensive adenopathy including patients with large (> 5 cm), usually inoperable tumours and node positive disease [1]. It is a common presentation worldwide but is of special concern in developing countries with limited breast cancer awareness and efficient population screening programs. For instance, in Serbia over 4500 new breast cancer cases are diagnosed each year, with as many as 30% presenting as late stage initially inoperable LABC.

A multimodal approach including systemic therapy, radiotherapy and surgery is usually applied in the treatment of LABC [2]. Currently, neoadjuvant systemic therapy (CHT) is usually administered to downstage the tumour for breast-conserving surgery, while preoperative radiotherapy (pRT) is often indicated if there is no objective reduction of tumour volume after the administration of neoadjuvant (CHT). Radiation therapy (RT) is frequently used in various modalities for treatment of breast cancer of different stages including LABC, and large meta-analyses of multiple randomised trials demonstrated clear long-term benefit both in terms of locoregional control and reduced mortality in breast cancer patients treated with radiotherapy after breast conserving surgery and after radical mastectomy [3–5]. Given the rarity of this treatment modality only a few studies looked into the effects of preoperative radiotherapy (pRT) in combination with breast conserving surgery on locoregional recurrence and overall survival reporting similar results compared to protocols involving neoadjuvant chemotherapy without irradiation [6–9]. However, the molecular basis of tumour sensitivity to radiotherapy is complex, and at present, there are no conclusive biomarkers in clinical use to predict if a patient will, in fact, benefit from radiotherapy.

The concept of personalised medicine has been successfully implemented in medical oncology for over a decade with several biomarkers approved for clinical use. The same principle could be applied to radiation oncology to achieve better clinical responses to radiotherapy, lower radio-toxicity and avoid overtreatment [10, 11]. To achieve this goal there is a clear need to develop biomarkes specific for breast radiotherapy. Hovewer, most of the studies in radiation oncology have been limited to the study of biomarkers not necessarily chosen based on their specificity to radiotherapy. Molecular subtypes have shown limited predictive estimation of RT efficacy and have been potentially confounded by adjuvant systemic therapy [11–13]. Several studies aimed to identify a radiosensitivity molecular signature in breast cancer by studying changes in gene and protein expression in response to radiation in cellular and animal model systems. These included determination of cellular radiosensitivity defined by survival fraction at 2 Gy [14], clonogenic doubling time, hypoxic fraction, or clonogenic number [15], with some of these multigene signatures having been validated in retrospective studies in solid tumours including breast cancer [16–18].

Although these studies provided valuable insights into cellular radiation response, the breast tissue has a complex microenvironment composed of several interacting cell types and extracellular molecules that may affect tumour response. The ideal model system for researching the breast tumour response to radiation therapy and evaluating the predictive value of markers is the preoperative setting [19, 20]. Detection and characterization of biomarkers in the patient’s tumour biopsies with known clinical response, before and after radiation therapy could select the group of patients with worse response to radiotherapy, to facilitate the choice of more efficient treatment, avoid overtreatment and consequentially reduce associated healthcare costs.

Leveraging a cohort of LABC patients treated with pRT without neoadjuvant or concomitant CHT (IORS LABC cohort) we determined baseline molecular differences between radio-resistant and radio-responsive breast tumours and identified putative predictive biomarkers of response to pRT. In our previous study on this cohort [21] we have shown that the extent of the clinical response to pRT in LABC is predictive of overall survival. Here, we analysed global gene expression patterns and biological pathways associated with differential response to pRT in a discovery subset of the IORS LABC cohort. Selected biomarkers were validated by an orthogonal assay (qPCR) in an independent validation set of pRT IORS LABC samples, and in the postoperative setting using an external microarray dataset (Erasmus cohort) consisting of breast cancer patients treated only with radiotherapy following surgery.

## Methods

### Patient cohorts

#### IORS LABC cohort

**Fig. 1:**
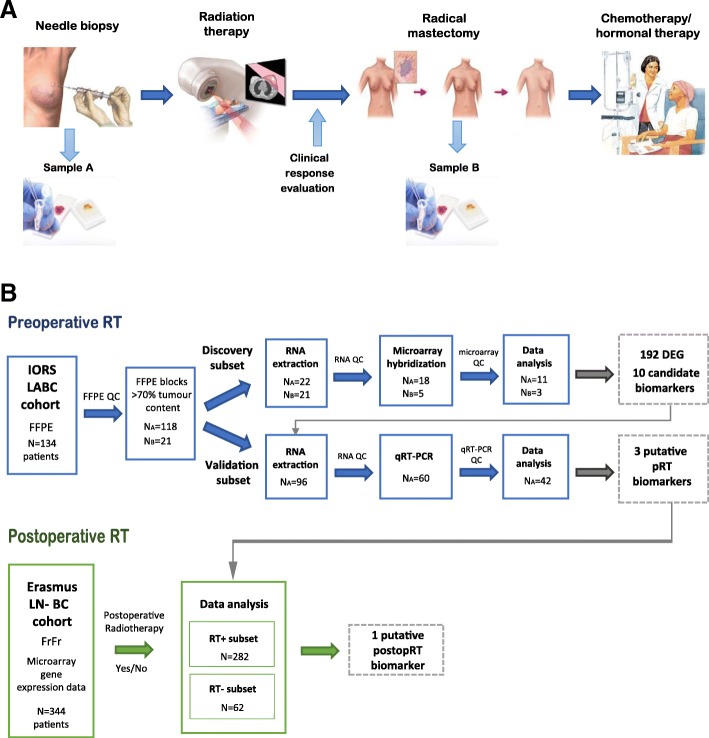
**a** Infographic summarizing treatment protocol and sample collection of locally advanced breast cancer patients treated at the Institute of Oncology and Radiology of Serbia between 1997 and 2000 (IORS-LABC cohort). The IORS-LABC cohort included 134 patients who had initial biopsy taken before any treatment, followed by radiotherapy and radical mastectomy. Exceeding tumour material was formalin-fixed and paraffin embedded (FFPE) and stored at room temperature. **b** Flowchart representing the study outline for sample processing, quality control, data analysis and biomarker validation. FFPE samples were review by a pathologist to select those with > 70% of tumour material, retaining 118 pre-RT biopsy tumour samples (**a**) and 21 post-RT tumour samples (**b**). These samples were split into discovery (N_A_ = 22 pre-RT and N_B_ = 21 post-RT) and validation (N_A_ = 96) subsets. After quality control of extracted RNA only 23 samples from the discovery subset were selected for microarray hybridization (N_A_ = 18 and N_B_ = 5), of which only 14 (N_A_ = 11 and N_B_ = 3) passed data quality control. Out of 96 pre-RT samples designated for validation, only 60 had passed RNA quality control and were used for qRT-PCR. Of those, 42 samples passed data quality control and were retained for the downstream analysis

This retrospective cohort included 134 patients with locally invasive non-inflammatory breast cancer (LABC) (93 patients stage III-a, and 41 patients III-b) treated with pRT between 1997 and 2000, delivering 45Gy in 15 fractions every second day alternately to the breast and regional lymph nodes, followed by radical mastectomy and adjuvant chemo and/or hormonal therapy (Fig. 1a). The median follow-up was 74 months (4–216) counted from the breast cancer diagnosis until last check-up or death from any cause. Five-year overall survival was 56% and 5-year disease free survival was 39.2%. Tumour biopsy was taken both prior to radiation treatment to obtain a radiotherapy-naïve sample (series A), and after RT and radical mastectomy before adjuvant treatment (series B) stored as formalin-fixed paraffin-embedded (FFPE) tissue samples. Clinical response to pRT in the breast was defined per RECIST criteria [22]. All patients gave their informed written consent for the use of residual tissue for research. The study was approved by the Institute of Oncology and Radiology Ethical Review Board for human studies. An overview of the patients’ clinicopathological characteristics is summarised in Additional file 1: Table S1.

#### Erasmus breast cancer cohort

For biomarker validation, we have used a previously published clinical data set from the Erasmus cohort (GSE2034 and GSE5327). This cohort includes 344 lymph-node negative breast cancer patients treated at the Erasmus Medical Center (Rotterdam, Netherlands) from 1980 to 1995, who had not received neoadjuvant nor adjuvant systemic treatment [23]. Primary treatment was breast-conserving surgery or modified radical mastectomy, and 87% of the patients received postoperative RT. Early metastasis was defined as distant recurrence in the first 5 years following completion of primary treatment.

#### RNA extraction from FFPE tissue

Tumour tissue sections were stained with H&E and examined by a pathologist. Tumour tissue was macrodisected prior to RNA extraction to ensure there was > 70% tumour content. Total RNA was extracted from 3 to 5 10 μm thick FFPE tissue sections using RNeasy FFPE Kit (Qiagen) with an 18-h Proteinase K tissue digestion step. RNA quantity and purity were assessed by BioSpec-nano (Shimadzu Scientific Instruments).

#### Microarray hybridization and data analysis

Agilent SurePrint G3-Hmn-GE-v.2-8x60K Microarray platform was used for gene expression profiling following Agilent Gene Expression FFPE Workflow. Raw data pre-processing and quality control was performed using R version 3.0.1 and R/Bioconductor packages ‘limma’, ‘ffpe’ and ‘ArrayQualityMetrics’. Data were deposited in the GEO database under the GSE101920 accession number. Hierarchical average linkage clustering was performed using Cluster 3.0 and visualised using JavaTreeView [24]. Differential expression analysis was performed using the POMELO II tool applying moderated t-test [25]. The estimated significance levels were corrected for multiple hypotheses testing using Benjamini and Hochberg False Discovery Rate (FDR) adjustment [26]. The ranked target list of the differentially expressed genes was subjected to pathway enrichment analyses using Ingenuity Pathway Analysis software (Ingenuity Systems). Significantly enriched gene networks and canonical pathways based on the curated IPA database KEGG, Biocarta, and Reactome, were identified as previously described [15]. Methods are described in detail in Additional file 2: Supplementary Methods.

#### Quantitative RT-PCR and statistical analysis

Applied Biosystems High Capacity cDNA Reverse Transcription Kit was used for preparing cDNA from 200 ng RNA. Quantitative RT-PCR was done on an ABI Prism 7300 (Applied Biosystems) using TaqMan® Gene Expression Assays and TaqMan® PreAmp Master Mix Kit (Life Technologies). All qPCR amplicons were designed to be less than 100 bp long and all assays were done in triplicate. Assay ID numbers are shown in Supplementary Table 4. Each plate included a HeLa cell line as inter-plate calibrator (IPC) and a non-template control (NTC). Average Ct values for each gene were standardised to IPC, dCt values were calculated relative to *ACTB* as a reference gene. Genes and samples with over 70% of missing values were excluded from further analysis, retaining 8 genes and 42 samples for further analysis. Undetermined values were set to the number of cycles performed (Ct = 45). Data was log2 transformed and differences in gene expression levels between groups were tested using Student t-test and corrected for multiple testing with FDR set to 20%.

#### Erasmus dataset processing and statistical analysis

Erasmus dataset was downloaded from GEO database and processed using *affy* R package. Distant metastasis free survival (DMFS) was defined as any distant recurrence within 5 years after the end of treatment. Survival curves were plotted with the Kaplan–Meier method and log-rank test was used to evaluate differences between groups defined by candidate gene expression status. Cutoff Finder was used to determine the optimal cutpoint for gene expression dichotomization based on the log-rank test minimum *P*-value approach [27]. Hazard ratios were estimated using the Cox proportional hazards model, stratified by RT status in a univariable and multivariable analysis. Pearson’s χ^2^ test was used to check for unbalanced distribution of clinico-pathologcal varables (ER, PR, T-stage and age categories) in subgroup analysis (Additional file 3: Table S2). Measures of biological interaction were determined both on additive scale and multiplicative scale [28, 29]. Stata command icp was used for calculating 3 different measures of interaction contrast on an additive scale: relative excess risk due to interaction [RERI], attributable proportion [AP] and synergy index [S] as described in [30]. Multiplicative interaction was assessed by including an interaction term with main effects in the Cox proportional hazard model. Statistical calculations were performed using STATA version 11.2 (StataCorp). All reported *p*-values were two-sided with a 0.05 significance level.

## Results

### Molecular signature of radiosensitivity in LABC tumour samples

To gain a better understanding of the molecular response to radiotherapy independently of systemic treatment and to identify a baseline transcriptional signature of radiosensitivity between radio-responsive and non-responsive tumours, we have analysed radiotherapy-naïve LABC tumour biopsies and post radiotherapy mastectomy samples by gene expression profiling. A total of 43 FFPE tumour samples (N_A_ = 22, N_B_ = 21) from the patient cohort was randomly selected maintaining balanced group representation of clinical response (CR, PR, SD) and other clinicopathological characteristics (Additional file 4: Table S1). Of those, due to the low concentration or purity of the RNA extracted from FFPE only 23 samples were selected for microarray hybridization. Following stringent microarray quality control 14 samples (N_A_ = 11, N_B_ = 3) were included in subsequent data analysis (Additional file 4: Figure S1), comprising of 8 patients with stable disease designated as non-responders (NR) and 6 patients which experienced either complete (2 pts) or partial clinical response (4 pts) to pRT, classified as responders (R) (Fig. 1b).

**Fig. 2:**
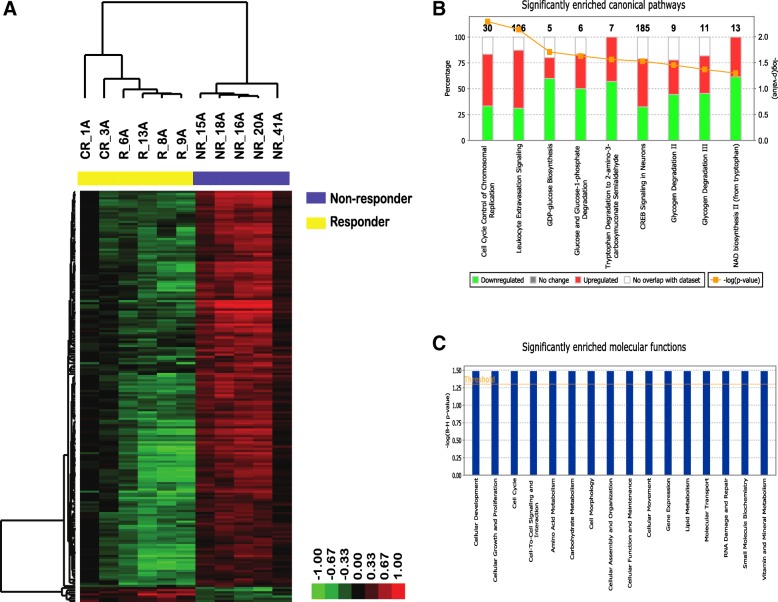
**a** Supervised average linkage hierarchical clustering of 11 preRT FFPE tumour samples from locally advanced breast cancer (LABC) patients treated with preoperative radiotherapy (pRT) over 192 significantly differentially expressed gene transcripts. **b** Significantly enriched Canonical pathways and (**c**) Molecular functions identified by Ingenuity Pathway Core Analysis

Although unsupervised clustering analysis over top 20% most variable transcripts revealed separation between non-responders and responders (Additional file 5: Figure S2), not enough post-RT samples were left after QC to draw relevant conclusions regarding changes in pRT response between pre-RT and post-RT biopsies. Therefore, to study intrinsic differences underlying differential response to radiotherapy in the neoadjuvant setting, we have analysed only pre-RT tumour samples (N_A_ = 11) to look for differences in baseline transcriptional profiles between responders (R) and non-responders-(NR). We identified 192 significantly differentially expressed mRNA transcripts (> 2-fold change and FDR < 0.1), including 89 annotated protein coding genes (PCG) and 78 long non-coding RNAs (lncRNAs) (Additional file 6: Table S3). Of those, only 7 genes were found to be upregulated while the rest of the genes (185) were downregulated in radio-responsive tumours (R) (Fig. 2a**)**. The top 20 differentially expressed PCG are listed in Table 1**.**

**Table 1:** Top 20 significantly differentially expressed protein coding genes between pRT responsive and non-responsive tumor samples

#	Gene Symbol	Gene Name	FDR-adjusted q-value	Fold Change	Super-Pathways
1	ST3GAL4	ST3 beta-galactoside alpha-2,3-sialyltransferase 4	*0.03715* ^##^	**0.10**	*protein glycosylation*
2	C6orf105 (ADTRP)	chromosome 6 open reading frame 105 (Androgen-Dependent TFPI-Regulating Protein)	*0.03715* ^##^	**0.17**	No Data Available
3	RAP1GAP2	RAP1 GTPase activating protein 2	*0.03781* ^##^	**0.18**	*Immune System*
4	A1CF	APOBEC1 complementation factor	*0.03874* ^##^	**0.17**	*mRNA Editing* and *Processing of Capped Intron-Containing Pre-mRNA*
5	MAP3K4	mitogen-activated protein kinase kinase kinase 4	*0.03874* ^##^	**0.25**	*MAPK signaling pathway*
6	CHD5	chromodomain helicase DNA binding protein 5	*0.05133* ^#^	**0.15**	*ATP-dependent helicase activity*
7	LAS1L	LAS1-like (*S. cerevisiae*)	*0.05209* ^#^	**0.19**	*biogenesis of the 60S ribosomal subunit.*
8	DEFB128	defensin, beta 128	*0.05781* ^#^	**0.29**	*Immune System*
9	ENHO	energy homeostasis associated	*0.05781* ^#^	**0.23**	*metabolism*
10	CECR9	cat eye syndrome chromosome region, candidate 9 (non-protein coding)	*0.05781* ^#^	**0.20**	*unknown*
11	IDO1	indoleamine 2,3-dioxygenase 1	*0.05781* ^#^	**0.20**	*Tryptophan metabolism*
12	LRRC55	leucine rich repeat containing 55	*0.05788* ^#^	**0.14**	*ion channel*
13	ROGDI	rogdi homolog (Drosophila)	*0.05788* ^#^	**0.26**	*unknown*
14	KRT25	keratin 25	*0.05796* ^#^	**0.22**	*cytoskeleton*
15	LAMA4	laminin, alpha 4	*0.05798* ^#^	**0.25**	*Focal Adhesion, ECM-receptor interaction*
16	PLA2G2C	phospholipase A2, group IIC	*0.05798* ^#^	**0.15**	*alpha-Linolenic acid metabolism and Glycerophospholipid biosynthesis*
17	CCDC114	coiled-coil domain containing 114	*0.05798* ^#^	**0.25**	*cell motility*
18	CNGB1	cyclic nucleotide gated channel beta 1	*0.05798* ^#^	**0.27**	*cAMP binding and intracellular cAMP activated cation channel activity*
19	PRSS53	protease, serine, 53	*0.05798* ^#^	**0.30**	*serine-type endopeptidase activity*
20	GSG1	germ cell associated 1	*0.05798* ^#^	**0.22**	*RNA polymerase binding*

*FDR* – false discovery rate; Fold change is shown on a linear scale

^#^*q*-value < 0.1; ^##^*q*-value < 0.05

To elucidate which biological processes and signalling pathways are associated with differential response to pRT, we have applied a gene set enrichment analysis using Ingenuity Pathways Analysis software. The bulk of differentially expressed genes were organised in two top scoring gene networks: lipid metabolism, molecular transport and small molecule biochemistry (Network 1) and cell cycle, DNA replication, recombination, and repair (Network 2) (Additional file 7: Figure S3). Specifically, differentially activated canonical pathways (Fisher’s test *p* < 0.05) between pRT responders and non-responders, included cell cycle control of chromosomal replication, and pathways related to glucose metabolism and de novo NAD biosynthesis (Fig. 2b). Similarly, molecular functions significantly enriched within the list of differentially expressed genes included cellular development, cell growth and proliferation, cell cycle and functions related to cell morphology, movement, assembly and organisation in addition to metabolic processes (Fig. 2c).

### Candidate gene validation by qRT PCR

Even after controlling for false discovery rate, due to the small size of the discovery cohort and technical challenges related to FFPE-based microarray hybridization, several associations with the outcome of interest may have occurred due to chance alone. Therefore, we proceeded to validate a selected panel of genes in an independent set of pre-RT tumour samples (N_A_ = 60) using an orthogonal assay (qRT-PCR). Ten genes (*CHEK2, XRCC2, MCM6, MAP3K4, MMP14, APOA1, WHSC1L1, IDO1, ST3GAL-4* and *A1CF*) were selected for validation based on the significance threshold, high expression in tumour tissue and plausible biological function. Samples in which over 70% of assays have failed were discarded, retaining 42/60 samples for statistical analysis (Fig. 1b). Considering the cost of missing a potentially interesting gene (false negative) versus the low cost of further external validation to discard any false positive calls, we decided to use a lax False Discovery Rate of 20%. Differential gene expression between responders (N_R_ = 30) and non-responders (N_NR_ = 12) was observed for *APOA1*, *MAP3K4* and *MMP14* genes with over 2-fold downregulation in pRT-responsive tumours (Table 2).

**Table 2:** Gene expression analysis by qRT-PCR in an independent subset of 42 LABC tumor samples

#	Gene Symbol	Amplicon Length	Responders (R)(*n* = 30)		Non-responders (NR)(*n* = 12)		p-value	Fold change
			mean	sd	mean	sd		
1	APOA1	**63**	5.99	1.96	7.55	1.37	0.0161**	0.34
2	CHEK2	109	−4.63	1.69	−4.18	1.06	0.390	0.73
3	IDO1	**106**	1.06	2.87	0.63	2.82	0.662	1.35
4	MAP3K4	89	−3.00	1.99	−1.78	1.28	0.0582*	0.43
5	MCM6	**109**	−5.12	1.98	−4.88	2.34	0.741	0.85
6	MMP14	92	7.85	2.85	9.61	2.03	0.0596*	0.30
7	ST3GAL4	**60**	−3.95	2.68	−3.38	3.32	0.566	0.67
8	WHSC1L1	**67**	−0.98	3.90	−1.16	4.61	0.979	1.13
9	XRCC2	**66**	−4.68	2.08	−4.04	2.13	0.455	0.64

Gene expression was determined by qRT-PCR in independent test set of 60 FFPE breast tumors, of wich 42 were retained for data anlysis. Represented data were interplate calibrated, normalized to B-Actin and log2 transformed. Normality was evaluated using Lilform test. p-values - level of significance according to the Student’s t-test or nonparametric Kolmogorov-Smirnov test (WHSC1L1 and ST3GAL4), *FC* fold change gene expression relative to ACTB between pRT responders (R) to nonresponders (NR) tumors measured by qPR-PCR; Fold change is shown on a linear scale

*p-value < 0.1; **p-value < 0.05

### External validation of candidate gene predictive value for radiotherapy response

**Fig. 3:**
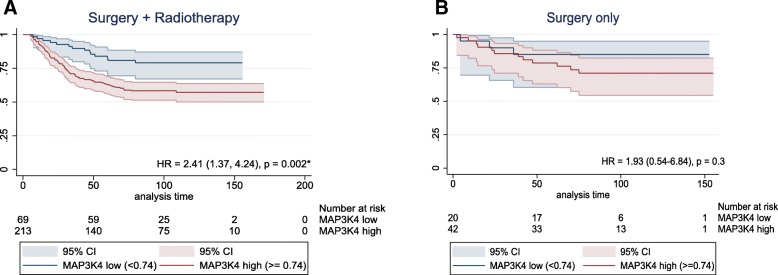
Association of distant metastasis-free survival with high (red) and low (blue) *MAP3K4* expression in Erasmus breast cancer dataset. **a** Kaplan-Meier survival estimates of 282 patients treated with surgery and RT (**b**) Kaplan-Meier survival estimates of 62 patients treated with surgery only

To evaluate whether expression of *APOA1*, *MAP3K4* and *MMP14* genes has an impact on patient survival in the postoperative setting, we analysed distant metastasis-free survival (DMFS) using a well-characterized cohort of 344 lymph node-negative breast patients undergoing surgical treatment, with or without postoperative radiotherapy (Erasmus cohort) (Fig. 1b). The Erasmus cohort was chosen to eliminate potential confounding effects of systemic therapy, and to asses weather these genes are radiation-specific (predictive) or not (simply prognostic) in a stratified analysis. Of the three genes tested in a subgroup analysis, only low *MAP3K4* expression (< 7.94) was significantly associated with better DMFS (HR = 2.41 [95%CI:1.37–4.24], *p* = 0.002) in 282 patients treated with both surgery and RT, but not for those 62 patients undergoing only surgery (HR = 1.93 [95%CI:0.54–6.84], *p* = 0.309) indicating that the effect is specific for RT-treated patients (Fig. 3). After controlling for age, steroid receptor status, T-stage and menopause status as potential confounders in a multivariable analysis MAP3K4 remained an independent predictor of DMFS (HR = 2.54, [95%CI:1.42–4.55], p = 0.002) in RT-treated patients. (Table 3). To check for the presence of biological interaction between *MAP3K4* levels and radiotherapy, we calculated hazard ratios for each category combination and summary measures of effect modification on both multiplicative and additive scale. There was no evidence of statistical interaction on a multiplicative scale, while the combined effect of *MAP3K4* levels and radiotherapy on additive scale exceeded that of each exposure alone with a 0.91 relative excess risk due to interaction [RERI] (Table 4).

**Table 3:** Multivariable Cox regression analysis of distant metastasis free survival in 282 patients treated with radiotherapy and surgery

Variable	Reference vs. level	Hazard Ratio	(95% CI)	*p*-value
*MAP3K4 level*	*(low* vs. *high)*	2.54	(1.42, 4.55)	0.002*
*ER/PR status*	*(ER+/PR+* vs. *ER-/PR+ or ER+/PR-, ER-/PR-)*	1.20	(0.94, 1.52)	0.146
*Age*	*(under 40* vs. *40–55, 56–70, over 70)*	0.81	(0.53, 1.24)	0.332
*Menopause*	*(pre-menopausal* vs. *postmenopausal)*	1.19	(0.58, 2.42)	0.639
*T-stage*	*(T1* vs. *T2, T3, T4)*	1.08	(0.75, 1.55)	0.697

*p-value < 0.01

**Table 4:** Hazard ratios for distant metastasis free survival with 95% CI in 344 lymph node negative breast cancer patients with measures of effect modification

*MAP3K4* level	Radiotherapy	Hazard Ratio	(95% CI)	p-value
low	no	Reference = 1		
low	yes	1.33	(0.38, 4.63)	0.652
high	no	1.97	(0.56, 6.97)	0.294
high	yes	3.21	(1.02, 10.15)	0.047*

Measure of effect modification on additive scale: RERI (95% CI) 0.91 (−0.56, 2.39); *P* = 0.226

Measure of effect modification on multiplicative scale: ratio of HRs (95% CI) 1.23 (0.31, 4.90); *P* = 0.773

## Discussion

Treatment of LABC continues to be challenging with patients being at increased risks of locoregional recurrence, distant metastasis and reduced quality of life. Breast radiotherapy was shown to be effective in the locoregional control and provided benefit in distant metastasis-free survival and for downstaging the tumour in the preoperative treatment of LABC. However, not all patients achieve a satisfying response to radiotherapy. Clinically, a tumour is considered radioresistant when irradiation is unable to reduce its volume or when a recurrence occurs after a possible regression. Thus, it would be beneficial to identify biomarkers predictive of initial response to pRT that could be useful to predict clinical outcome in RT treated patients.

Here we explored gene expression profiles in pre- and post-RT tumour biopsies of LABC samples with different clinical response to pRT. However, after microarray QC there were too few pre- and post-RT matched samples to be able to draw any statistically significant conclusions regarding changes induced by pRT. Therefore, we focused our further analysis on pre-RT biopsies only, to establish baseline differences in transcriptional profiles between patients achieving either complete or partial clinical response and those who did not respond to pRT. Among 192 significantly differentially expressed transcripts, only 89 corresponded to known protein coding genes and to 78 lncRNA. Although, lncRNAs were shown to have important roles in a broad range of biological processes such as at the level of post-transcriptional processing and transcriptional gene silencing [6, 7] their biological function in breast cancer, and especially in relation to radiation therapy remains unknown.

Using IPA analysis, we explored which gene networks and signalling pathways are conferring radiation resistance/sensitivity in LABC tumour samples. Looking at protein interactions, most of the genes were organised in two major networks, one constituted of genes involved in lipid metabolism, molecular transport and small molecule biochemistry and the second related to cell cycle, DNA replication, recombination, and repair. These results further emphasise the importance of the cell cycle and proliferation state on cellular radiosensitivity, in line with the current body of knowledge [31, 32]. Interestingly, when focusing on specific canonical pathways in addition to cell cycle control of chromosomal replication, pathways related to glucose metabolism and de novo NAD biosynthesis were significantly overrepresented. This profile potentially reflects a comparatively activated metabolic pathway for de novo synthesis of NAD+ in radio-resistant tumours. In addition to its numerous functions in redox reactions, NAD+ is a sole substrate for the activity of PARP enzyme in the repair of DNA single-strand brakes [33, 34]. Several inhibitors of *IDO1* gene, a rate-limiting enzyme for the NAD+ de novo synthesis from tryptophan found to be downregulated in radiosensitive LABC tumours, have shown a radiosensitising effect in in pre-clinical studies, making it an attractive target for drug-radiation combination therapy [35–37].

The second aim of the study was to identify potential biomarkers of response to radiotherapy. To this end, we selected 10 genes to be validated by an orthogonal assay in an independent subset of LABC tumours. Only 3 genes (*APOA1*, *MAP3K4* and *MMP14*) were confirmed to be downregulated in radiosensitive tumours in the preoperative setting. These markers were further validated in an independent dataset of breast tumours treated only with mastectomy with or without postoperative radiotherapy. Only *MAP3K4* gene was found to independently and specifically predict DMFS in radiotherapy-treated patients. Patients with high levels of *MAP3K4* treated with RT had shorter DMFS than those with low *MAP3K4* levels, due to an adverse interaction of high *MAP3K4* expression with RT. *MAP3K4* is a member of first layer of kinases of the MAPK signalling pathway that is activated by a variety of stimuli, including ionizing radiation, to mediate activation of transcription factors controlling differentiation, proliferation, cell growth and survival [38]. Increased expression of *MAP3K4* in breast tumours may confer radioresistance through augmented signalling for RT-induced DNA damage repair through G2 arrest thus aiding survival of irradiated cancer cells [39]. Therefore, detecting the levels of *MAP3K4* expression in breast tumours, may be useful for the prediction of response to RT for tumour downstaging for breast conserving surgery in LABC. Furthermore, inhibition of Ras-Raf-MEK-ERK cascade was shown to increase radiosensitivity both in in vitro as well as in vivo studies rendering MAPK signalling as an attractive radiosensitising target [40–42].

The main limitation of this study is a small sample size of the discovery cohort resulting from suboptimal RNA extracted from FFPE material, that may have led to inflated type I error. To mitigate this effect, we performed validation in larger independent series using an orthogonal assay. However, the limitation of the qPCR method using FFPE tissues is that it is sensitive to degraded DNA thus not all genes could have been detected in all patients’ samples. Despite these limitations, the value of these results lies in the use of LABC tumour samples exposed only to RT without systemic therapy administered either previously or concomitantly with RT, and further validation of candidate genes’ effect on DMFS in a large independent cohort devoid of any confounding effects of systemic therapy.

## Conclusions

In summary, this study provides a novel insight into the underlying biology of intrinsic breast tumour radioresistance and points to genes and pathways that may be targeted to increase radiosensitivity. Additionally, we identified a putative radiotherapy-specific biomarker of response, *MAP3K4* that warrants further mechanistic studies and validation in randomized prospective cohorts to optimise patient selection and treatment planning.

## Additional files

Additional file 1: **Figure S1.** Quality assessment and control for FFPE microarray expression data. (PDF 75 kb)

Additional file 2: **Figure S2.** Unsupervised average linkage hierarchical clustering over top 20% most variable genes of 14 FFPE tumour samples from locally advanced breast cancer (LABC) patients treated with preoperative radiotherapy (pRT). (PDF 3341 kb)

Additional file 3: **Figure S3.** Top scoring gene networks identified by Ingenuity Pathway Core Analysis. **A)** Network 1: lipid metabolism, molecular transport and small molecule biochemistry **B)** Network 2: cell cycle, DNA replication, recombination, and repair. (PDF 365 kb)

Additional file 4: **Table S1.** Clinico-pathological characteristics of the pre-radiotherapy LABC sample series included in the study. (XLSX 16 kb)

Additional file 5: **Table S2.** List of 192 differentially expressed transcripts between radio-responders and radioresistant pre-operative LABC samples. (XLSX 42 kb)

Additional file 6: **Table S3.** List of TaqMan Gene Expression Assays used for validation (XLSX 9 kb)

Additional file 7: **Supplementary Methods.** Detailed description of microarray hybridization, microarray data analysis, pathway enrichment analysis, validation of mRNA expression profiles by Q-RT-PCR and statistical analysis. (DOCX 25 kb)

## Abbreviations

cCD: Clinical complete response
CHT: Neoadjuvant systemic therapy
CI: Confidence interval
cPC: Clinical partial responder
cSD: Clinical stable disease
DMFS: Distant metastasis free survival
FDR: False discovery rate
FFPE: Formalin-fixed paraffin-embedded
GEO: Gene expression omnibus
HR: Hazard ratio
IORS: Institute for Oncology and Radiology of Serbia
IPA: Ingenuity Pathway Analysis
IPC: Inter-plate calibrator
LABC: Locally advanced breast cancer
NR: Non-responder
NTC: Non-template control
pRT: Preoperative radiotherapy
qPCR: Quantitative polymerase chain reaction
R: Responder
RT: Radiotherapy
