# Supplementary figures and images for: Molecular signature of response to preoperative radiotherapy in locally advanced breast cancer
Source: Radiat Oncol. 2018 Oct 1;13:193. doi: 10.1186/s13014-018-1129-4 (PMC6167820; doi:10.1186/s13014-018-1129-4)

Supplementary Figure 1

**Rank correlation to median pseudochip  
grouped by IQR, lowest to highest**

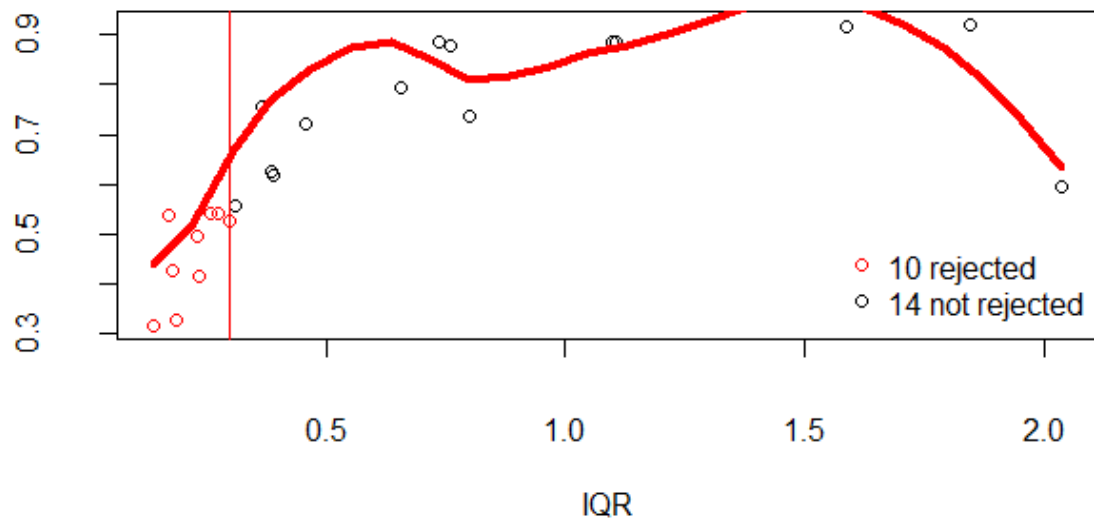

Supplement: Supplementary file 1 — Figure S1. Quality assessment and control for FFPE microarray expression data. (PDF 75 kb) [file 13014_2018_1129_MOESM1_ESM.pdf]

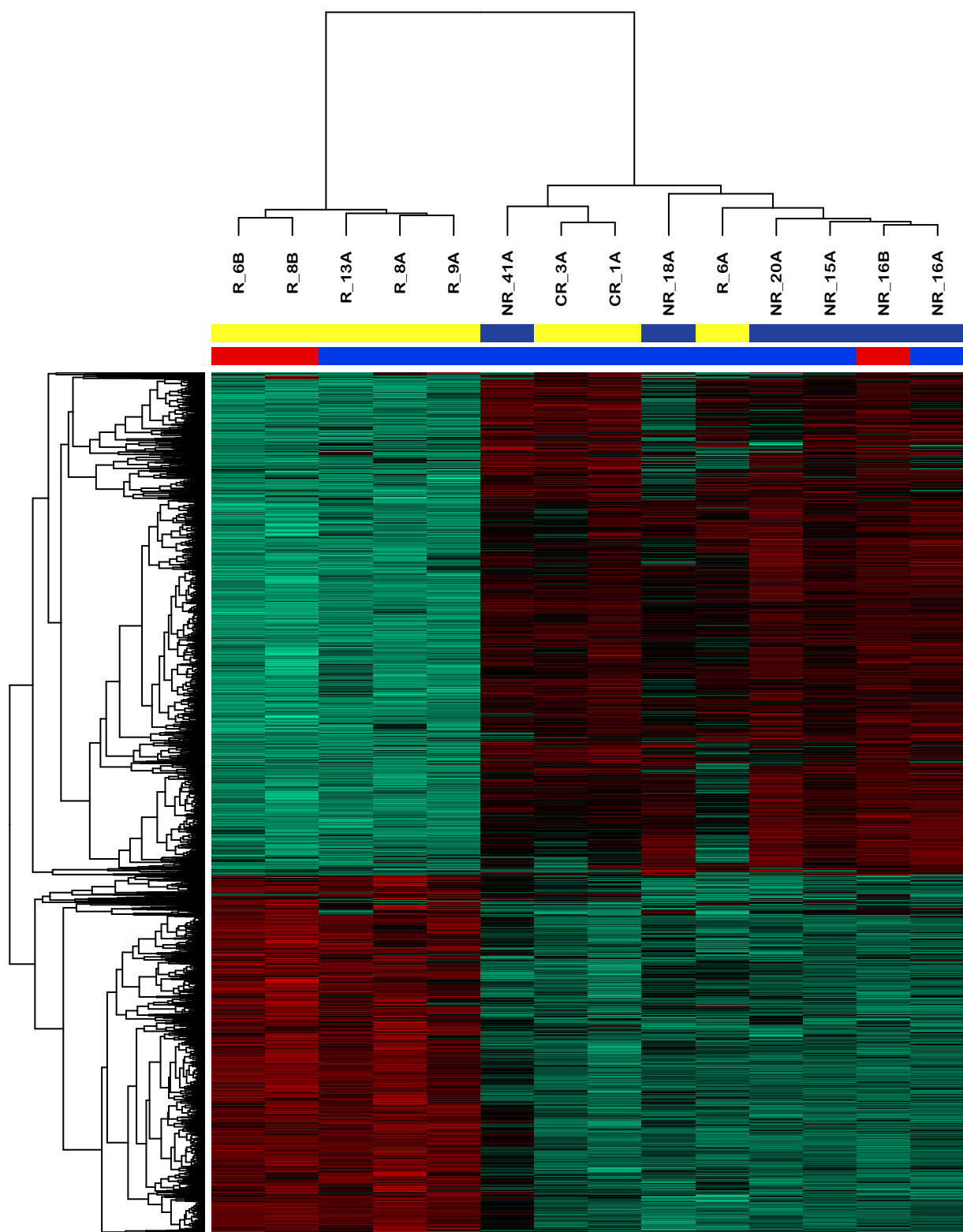

Supplement: Supplementary file 2 — Figure S2. Unsupervised average linkage hierarchical clustering over top 20% most variable genes of 14 FFPE tumour samples from locally advanced breast cancer (LABC) patients treated with preoperative radiotherapy (pRT). (PDF 3341 kb) [file 13014_2018_1129_MOESM2_ESM.pdf]
