# Supplementary material for: Molecular signature of response to preoperative radiotherapy in locally advanced breast cancer
Source: Radiat Oncol. 2018 Oct 1;13:193. doi: 10.1186/s13014-018-1129-4 (PMC6167820; doi:10.1186/s13014-018-1129-4)
Supplement: Supplementary file 7 — Supplementary Methods. Detailed description of microarray hybridization, microarray data analysis, pathway enrichment analysis, validation of mRNA expression profiles by Q-RT-PCR and statistical analysis. (DOCX 25 kb) [file 13014_2018_1129_MOESM7_ESM.docx]

### Supplementary Methods

### Microarray hybridization

Agilent SurePrint G3 Hmn GE v.2 8x60K Microarray covering 27,958 Entrez Genes and 7,419 lincRNAs was used for gene expression profiling. The hybridization procedure was performed according to Agilent Gene Expression FFPE Workflow. In brief, 300 ng of total RNA was reverse transcribed to generate double-stranded cDNA library using Transplex Whole Transcriptome Amplification (WTA) Kit (Sigma) and HotStarTaq Plus DNA Polymerase (QIAGEN), followed by purification using QIAquick PCR Purification Kit (QIAGEN). One microgram of the amplified WTA library was further labelled with ULS-Cy3 dye for 30 minutes at 85˚C using Genomic DNA ULS Lab Kit (Agilent) and Cy3-labeled samples were cleaned up using Agilent-KREApure columns. The puriﬁed, Cy3-labeled samples were combined with Agilent 10x Blocking Agent and Agilent 2x Hi-RPM Hybridization Solution. Agilent-CGHblock (a component of the Genomic DNA ULS Labeling Kit) was added and samples were denatured at 95˚C for 3 minutes prior to array hybridization on the SurePrint G3 Hmn GE v.2 8x60K microarray, for a period of 17h at 65ºC. Washing was performed according to the procedures outlined in the Agilent One-Color Microarray-Based Gene Expression Analysis manual (Version 5.7). Washed microarray slides were scanned using Agilent SureScan Microarray Scanner (Agilent Technologies, Santa Clara, CA, USA). Images were processed using Agilent Feature Extraction v.11 software using default settings.

### Microarray data analysis

Raw data preprocessing and quality control of IORS LABC cohort was performed using R version 3.0.1 and R/Bioconductor packages 'limma', 'ffpe' and 'ArrayQualityMetrics'. We filtered raw data to remove control probes and low expressed probes (less than 10% brighter than the negative controls), retaining 50751 probes. Quality assessment and control for FFPE microarray expression data was performed using 'arrayQualityMetrics' package [[1](#_ENREF_1)]. Ten samples didn't pass the cutoff (0.3 R^2^) and were excluded from further analysis. Filtered data were background subtracted using the normexp method with offset set to 50 and normalized for inter-array comparison by scaling to the median intensity, and log2 transformed. Probes with over 70% of missing values were removed, otherwise, missing values were imputed using KNN=15. Replicate probes were averaged by median value, retaining a total of 42628 probes including 38739 annotated probes (24924 Entrez genes and 13815 ncRNAs). Normalised data were deposited in the GEO database under the GSE101920 accession number. Erasmus datasets GSE2014 and GSE5327 were downloaded from GEO database and processed using *affy* R package.

Hierarchical average linkage clustering using Pearson uncentered correlation coefficient over top 20% most variable genes was performed using Cluster 3.0 and visualised as heatmaps using JavaTreeView (Version 1.1.6r2, jtreeview.sourceforge.net). Data were centred on the mean of the cluster means for patients. Normalised data was filtered by the standard deviation to retain top 20% most variable genes. Differential expression analysis was performed using linear models moderated t-test (limma), implemented in the POMELO II tool from Asterias package [[2](#_ENREF_2)]. The estimated significance levels (unadjusted p-values) were corrected for multiple hypotheses testing using Benjamini and Hochberg False Discovery Rate (FDR) adjustment and q-values (adjusted p-values) were reported [[3](#_ENREF_3)]. Those genes with q-value <0.05 were selected as significantly differentially expressed.

### Pathway enrichment analysis

The ranked target list of the differentially expressed genes was subjected to pathway enrichment analyses using Ingenuity Pathway Analysis software (IPA, Ingenuity Systems, Redwood City, CA).

The ranked target list of the differentially expressed genes was interrogated for the enrichment of specific molecular functions, canonical pathways and molecular networks using Ingenuity Pathway Analysis software (IPA, Ingenuity Systems, Redwood City, CA). IPA combines the information from manually curetted Ingenuity Pathway Knowledge Base (IPKB), KEGG pathways, Biocarta and Reactome databases, to identify biological networks significantly over-represented in the gene expression data. Right-tailed Fisher's exact test was applied to determine the level of significance, and the p-value was displayed as a score, which is the negative logarithm of the p-value. A score of 6 indicates there is a 1 in 10^6^ chance that the given genes are grouped together due to chance alone.

Gene networks and canonical pathways representing key genes were identified using the curated IPA (Ingenuity Pathway Analysis) database according to KEGG, Biocarta, and Reactome, as previously described [15]. Fisher’s exact test and v2 test were used to select the significant pathway, and the threshold of significance was defined by the P-value and FDR.

### Validation of mRNA expression profiles by Q-RT-PCR

Applied Biosystems High Capacity cDNA Reverse Transcription Kit was used for preparing cDNA from 200 ng RNA. Quantitative RT-PCR was done on an ABI Prism 7300 (Applied Biosystems) using custom TaqMan® Gene Expression Assays with amplicons shorter than 110 bp and TaqMan® PreAmp Master Mix Kit (Life Technologies) with 20 ng cDNA input in 25 µl final volume for the pre-amplification reaction using pooled assays in equimolar concentration. Pre-amplified cDNA was diluted 20x and 4 µl were used for qPCR reaction in 20 µl final volume for 45 cycles. All assays were done in triplicate. Each plate included a HeLa cell line as inter-plate calibrator sample (IPC) and a non-template control sample (NTC). Undetermined values were set to the number of cycles performed (Ct=45). Average Ct values for each gene were standardised to IPC, dCt values were calculated relative to ACTB as a reference gene. Gene assays that had failed across >70% of samples were excluded from further analysis (A1CF). Samples in which >70% of assays failed were discarded, retaining 42 samples for further analysis.

### Statistical analysis

Shapiro-Wilk W test was used to evaluate if qPCR gene expression data followed the normal distribution. Differences in log2 transformed gene expression levels were tested using Student t-test for those with a normal distribution, otherwise, the non-parametric Mann-Withney-Wilcoxon test was used.

Cutoff Finder (molpath.charite.de) [[4](#_ENREF_4)] was employed to find the optimal cutpoint based on the log-rank test minimum P-value approach for grouping patients for survival analysis. Distant metastasis free survival was estimated with the Kaplan–Meier method and hazard ratios were estimated using the Cox proportional hazards model. All reported p-values were two-sided with a 0.05 significance level. Statistical calculations were performed using STATA version 11.2 (StataCorp, College Station, TX, USA) and R [[5](#_ENREF_5)].

[1] Kauffmann A, Gentleman R, Huber W. arrayQualityMetrics--a bioconductor package for quality assessment of microarray data. Bioinformatics. 2009;25:415-6.

[2] Bignell GR, Warren W, Seal S, Takahashi M, Rapley E, Barfoot R, et al. Identification of the familial cylindromatosis tumour-suppressor gene. Nat Genet. 2000;25:160-5.

[3] Benjamini Y, Drai D, Elmer G, Kafkafi N, Golani I. Controlling the false discovery rate in behavior genetics research. Behav Brain Res. 2001;125:279-84.

[4] Budczies J, Klauschen F, Sinn BV, Gyorffy B, Schmitt WD, Darb-Esfahani S, et al. Cutoff Finder: a comprehensive and straightforward Web application enabling rapid biomarker cutoff optimization. PloS one. 2012;7:e51862.

[5] Team. RDC. R: A Language and Environment for StatisticalComputing. In: R Foundation for Statistical Computing. Vienna A, editor.2014.
