# Supplementary material for: Molecular signature of response to preoperative radiotherapy in locally advanced breast cancer
Source: Radiat Oncol. 2018 Oct 1;13:193. doi: 10.1186/s13014-018-1129-4 (PMC6167820; doi:10.1186/s13014-018-1129-4)

Supplementary Figure 1

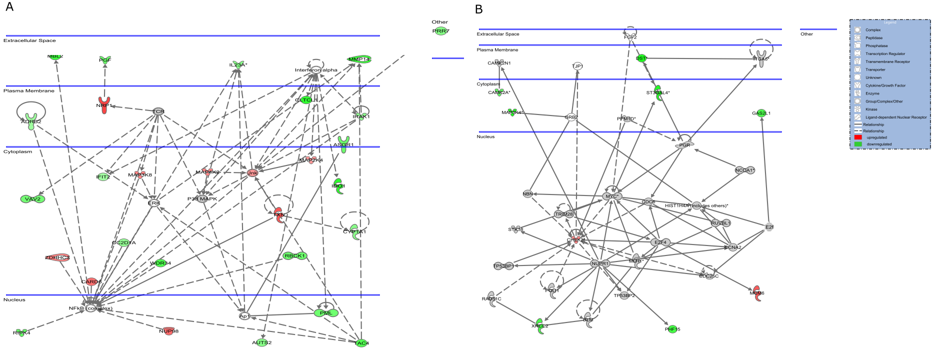

Supplement: Supplementary file 3 — Figure S3. Top scoring gene networks identified by Ingenuity Pathway Core Analysis. A) Network 1: lipid metabolism, molecular transport and small molecule biochemistry B) Network 2: cell cycle, DNA replication, recombination, and repair. (PDF 365 kb) [file 13014_2018_1129_MOESM3_ESM.pdf]
